# The worldwide frequency of MYO15A gene mutations in patients with non-syndromic hearing loss: A meta-analysis

**DOI:** 10.22038/IJBMS.2020.35977.8563

**Published:** 2020-07

**Authors:** Mahsa Farjami, Reza Assadi, Fahimeh Afzal Javan, Malihe Alimardani, Saeid Eslami, Sima Mansoori Derakhshan, Atieh Eslahi, Majid Mojarrad

**Affiliations:** 1 Department of Medical Genetics, Faculty of Medicine, Mashhad University of Medical Sciences, Mashhad, Iran; 2 Student Research Committee, Faculty of Medicine, Mashhad University of Medical Sciences, Mashhad, Iran; 3 Department of Education Development Center, Mashhad University of Medical Sciences, Mashhad, Iran; 4 Department of Genetics and Molecular Medicine, Faculty of Medicine, Mashhad University of Medical Sciences, Mashhad, Iran; 5 Pharmaceutical Research Center, Faculty of Pharmacy, Mashhad University of Medical Sciences, Mashhad, Iran; 6 Department of Medical Informatics, Faculty of Medicine, Mashhad University of Medical Sciences, Mashhad, Iran; 7 Department of Medical Genetics, Tabriz University of Medical Sciences, Tabriz, Iran; 8 Ibn Sina Medical Genetic Diagnostic Laboratory, Tabriz University of Medical Sciences, Tabriz, Iran; 9 Neurosciences Research Center, Tabriz University of Medical Sciences, Tabriz, Iran; 10 Medical Genetics Research Center, Faculty of Medicine, Mashhad University of Medical Sciences, Mashhad, Iran

**Keywords:** Autosomal recessive, Deafness, Meta-analysis, Mutation, MYO15A, Non-syndromic hearing loss, Prevalence

## Abstract

*MYO15A* is the third most crucial gene in hereditary sensorineural hearing loss after *GJB2* and *SLC26A4*. In the present study, we reviewed the prevalence of *MYO15A* mutations in patients with autosomal recessive non-syndromic hearing loss (ARNSHL). In this meta-analysis, we conducted a search of PubMed, Web of Science, Excerpta Medica Database, and Scopus, and identified the articles up to September 2019 without any time limit. Two investigators independently selected the relevant papers and extracted the required information. A total of 44 case-control and case series studies were considered, and 4176 patients and 3706 healthy individuals, as the control group, were included. The pooled frequency of *MYO15A *mutations between patients suffering from ARNSHL was calculated as 6.2% (95% CI: 4.9-7.8, *P*-value<0.001). There was heterogeneity between our studies (*P*-value<0.001, I2=58.1%); therefore, the random-effects model was utilized for analysis. Given the results, in many countries, the *MYO15A* gene has a significant contribution to hearing loss. Moreover, in several regions, specific dominant mutations in this gene have been reported. Therefore, the ethnic background should be considered to investigate the mutations of the *MYO15A* gene.

## Introduction

The incidence of hearing loss (HL), as the most common sensory defect in newborns, is about 0.1–0.2% (1). This disease is a multifactorial disorder, which is caused by environmental, genetic, or a combination of these factors (2). Genetic factors are the leading etiology of more than 50% of the HL cases (3). The autosomal recessive inheritance pattern is responsible for more than 80% of non-syndromic hereditary HL (ARNSHL) (4). According to the literature, more than 60 genes and 100 loci are associated with ARNSHL, the most significant of which are *GJB2*, *SLC26A4*, *MYO15A*, *OTOF*, and *CDH23*, respectively (5). 

The discovery of the disease-causing mutations in each gene in population leads to genetic diagnosis and counseling (6). Regarding the evidence, mutations in *MYO15A* are known to be some of the common causes of severe to profound ARNSHL (7). Mutations in this gene cause both pre- and post-lingual forms of progressive HL (4, 8). The role of the *MYO15A* gene in the development of HL was identified in a village of Bengkala, Bali, for the first time (9). 

So far, several studies have demonstrated that a total of 192 recessive variants of *MYO15A *(*DFNB3*) are associated with HL (10). *MYO15A* is found on chromosome 17p11.2, which contains the *DFNB3* locus consisting of 66 exons that encode the myosin-XV with 3,530 amino acids (4). Myosin-XV is a member of the superfamily of molecular motors. This protein generates the force that drives the mobility within cells by binding to cytoskeletal actin fibers (11). 

These molecular motors, involved in actin organization in the hair cells of the cochlea, are essential for the maturation of these cells and the formation of stereocilia by delivery of whirlin proteins to its top (12). Due to the large size of the gene and having many exons, simple techniques for detecting mutations are not compatible with this gene. As a result, the frequency and spectrum of the mutations of this gene in most populations are mostly unknown.

However, scientists detected causative DNA mutations in exons of this large gene by new sequencing methods such as targeted genome capture and massively parallel sequencing techniques (2). No hotspot region has been reported for this gene as yet; however, most of the mutations have been found in the motor domain of *MYO15A*, which is the most conserved domain located between exons 3 to 24 and is considered as the top priority to check (13).

So far, many studies have been carried out into the importance and prevalence of mutations in *GJB2* and *SLC26A* genes. Nevertheless, few studies were conducted systematically to investigate the importance of *MYO15A* in the incidence of HL. Few studies were performed to estimate the carrier frequency of the *MYO15A* gene and the prevalence of mutations in this gene as well. Accordingly, the aims of this study were defining these values, showing the mutation rate of this gene in different parts of the world, and signing common or founder effects of ethnic groups if they exist.

## Materials and Methods

***Search strategy***

We conducted a literature search in PubMed, Web of Science, Excerpta Medica Database, and Scopus for the English language studies published before September 2019. The search was based on the set of items provided by the Preferred Reporting Item for Systematic Review And Meta-Analysis (PRISMA) statement and was performed by two authors (M.F and M.A). This study is a systematic review of all publications of HL due to *MYO15A* gene mutations. The keywords included: (“MYO15A” or “myosin XVa” or “myoXVa” or “DFNB3”) and (“hearing impairment” or “hearing loss” or “deafness”). Moreover, all the citations of the retrieved articles were checked out to recognize additional potential data sources. 

***Inclusion criteria***

Two independent reviewers read the titles and abstracts of the retrieved articles and chose those had reported the prevalence of mutations in the *MYO15A* gene or adequate information for calculating the frequency. Disagreements were resolved by consensus discussions; when the debate did not conclude, the disagreement was escalated by the corresponding author.

The inclusion criteria entailed study populations of more than ten patients, the English language, and the examination of the entire length of the* MYO15A* gene to find mutations. Also, review studies and the studies with overlapping data sources or those that were performed on isolated populations were excluded. In case of required information absence, we directly contacted with the corresponding author to request information. In the case of the lack of response and essential data, the selected study was excluded, too.

***Data extraction and quality assessment***

The characteristics were gathered for each study included, first author’s name, the year of publication, number of subjects in the healthy and deaf groups, number of patients enrolled without *GJB2* mutation, ethnic and origin of the study population, used diagnostic methods, number of patients with *MYO15A* mutations, and frequency of *MYO15A* mutations. The quality of each study was evaluated based on the quality assessment checklist for prevalence studies (14).

The scale of this instrument ranges from low to high risk. It includes items such as selection bias, blinding method, instrument reliability and validity, data collection methods, non-response rates, and the case definition. According to this checklist, studies with zero to three, four to six, and seven to nine scores are considered as low, moderate, and high risk, respectively. 

***Statistical analysis***

The descriptive analysis of data of included studies and their quality control scores are presented in Table 1. The data obtained from all included studies were considered to measure the prevalence of *MYO15A* mutation and carrier frequency. 

Meta-analysis was conducted with Comprehensive Meta-Analysis software (ver. 2.2.064, Biostat, Englewood, NJ, USA). The populations of several included studies were combinations of healthy individuals and patients; therefore, the subgroups of controlled and non-controlled were defined. The random-effects model of analysis was selected based on the high I^2^ score. 

The studies in the controlled group were assessed for the meta-analysis of the carrier frequency. Publication bias in included studies was estimated by the funnel plot and Egger test. The geographic distributions of the patients with this gene mutation were demonstrated on the map; moreover, the regional prevalence of *MYO15A* mutations in patients with HL was calculated (Table 1 and Table 2).

## Results

***Included studies***

**Figure 1:**
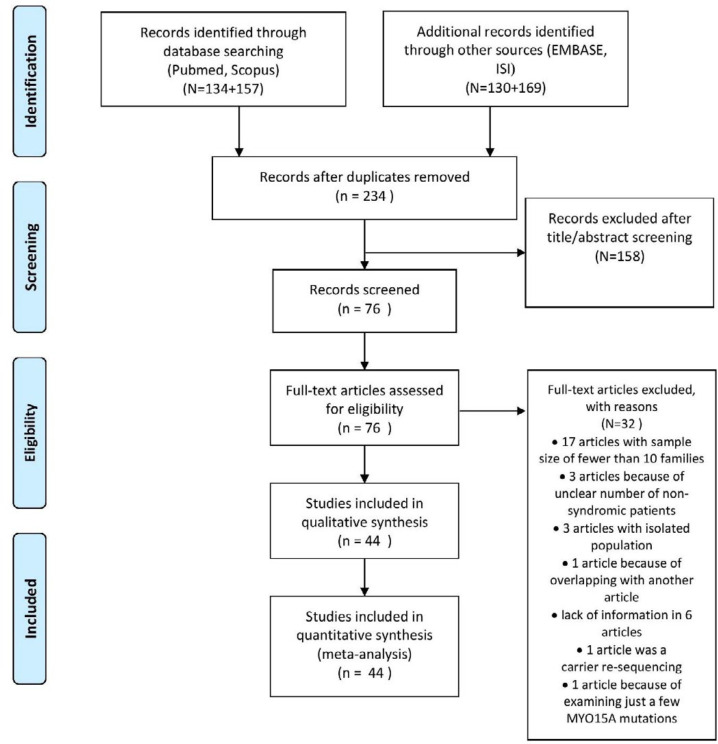
Preferred reporting items for systematic reviews and meta-analysis study selection flowchart

In the preliminary search, 590 records were identified from the selected database sources. After the exclusion of duplicated studies, a total of 234 records were obtained from various database resources and hand-searching journals. Finally, a total of 76 eligible records were selected by reading their titles and abstracts. The full texts of these studies were screened, and their qualities were determined; thereby, 32 records were excluded due to various reasons. Ultimately, 25 controlled and 19 non-controlled studies were considered in this systematic review (Figure 1).

Many studies were performed in Iran and Turkey. The sample number of included studies ranged from 10 to 854 individuals (4, 12). According to the quality assessment tool, 37 and 7 studies obtained low and moderate risk grades, respectively (Table 1). 

***Meta-analysis of the prevalence of MYO15A mutations among GJB2 negative ARNSHL patients***

**Figure 2:**
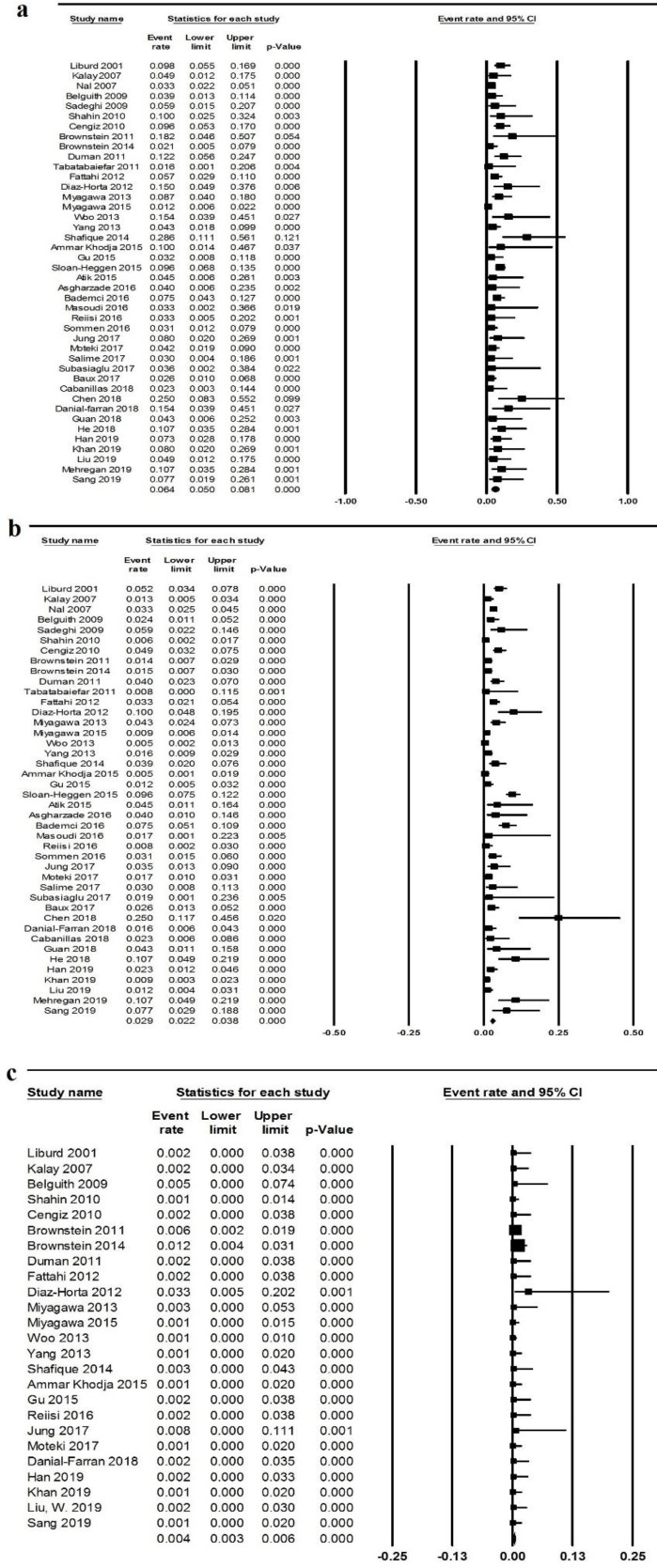
Forest plots of meta-analysis on the *MYO15A* mutation frequency using the random-effects model by Comprehensive Meta-Analysis software. a) The *MYO15A* mutation frequency among patients with autosomal-recessive non-syndromic hearing loss, who were GJB2 negative. All of the included studies had the same direction in this plot. The bars indicate 95% CI frequency of *MYO15A* mutations. I^2^=56%, *P*<0.001. The middle of the diamond shows the total prevalence. b) Forest plots of the overall prevalence of *MYO15A* mutation among all cases and controls. Chen’s study had the highest event rate. c) Forest plots on MYO15A carrier frequency in healthy controls. The number of controls in Diaz-Horta’s study was the lowest among 25 controlled studies; therefore, its CI95% was the widest. Moreover, no heterogeneity was found (I^2^=0%, *P*=0.75)

The prevalence of *MYO15A* mutations in deaf patients was estimated at 6.2% using 44 case series studies (95% CI: 4.9-7.8, *P*<0.001; Figure 2). Considering the 20% mutation rate of the *GJB2* gene, the frequency of *MYO15A* mutations was calculated as 4.9% among all patients with ARNSHL. The largest sample size belonged to the Miyagawa study (2015), and Ammar khodja study had the lowest. 

There was moderate heterogeneity (I^2^=58%, *P*<0.001); therefore, the random-effects model was applied to calculate this value. Because of the event rate that is always a positive amount, all of the included records were presented on the right side of the forest plots line.

Shafique study in Pakistan had the highest event rate, and the Miyagawa study (2015) in Japan had the lowest.

***Meta-analysis of the carrier frequency***

All of 25 controlled studies were entered into the carrier frequency analysis that contained 3706 healthy controls (the number of chromosomes was used for analysis due to the recessive inheritance pattern of the disease and considering the healthy carriers as the subjects of analysis) (Figure 2). No heterogeneity was found among our studies (I^2^=0%, *P*=0.75). 

Meta-analysis of the data demonstrated a carrier frequency of 0.4% (CI95%=0.3-0.6, *P*<0.001). Woo’s study had the highest number of controls, and the study conducted by Diaz-Horta had the lowest. In many studies, no mutation was detected in controls; therefore, most of the included records were presented in forest plots zero line.

***Meta-analysis of overall data***

For calculating the overall prevalence of *MYO15A* gene mutations, all controls and cases of 44 included studies were considered. Because of the heterogeneity of higher than 75%, the random-effects model was applied to analyze overall prevalence. A total of 7882 cases and controls were entered into the analysis, and the overall incidence was calculated as 3.6% (CI: 3.3-3.9, *P*<0.001; Figure 2). As shown in Figure 2, Chen 2018 study demonstrated the highest frequency.

**Figure 3:**
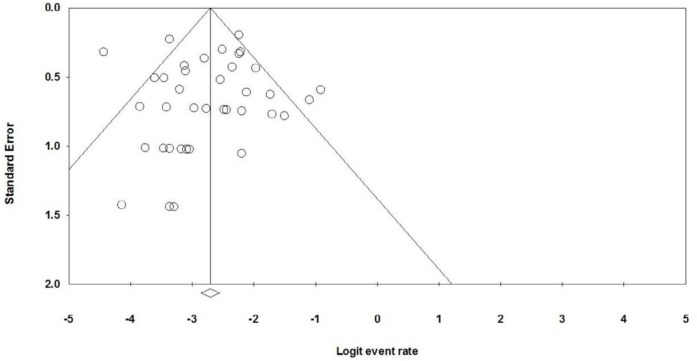
Funnel plots of included studies in meta-analysis to evaluate publication bias. Each hollow point represents a single study. The horizontal line displays natural logarithm of event rate

The Egger test and funnel plot analyzed the possibility of the publication bias in the included articles. According to the distribution of studies in Figure 3, the funnel plot is almost symmetrical. The Egger regression intercept was 0.063 (*P-value*=0.44). As a result, no significant publication bias was found in studies included in this meta-analysis.

**Figure 4:**
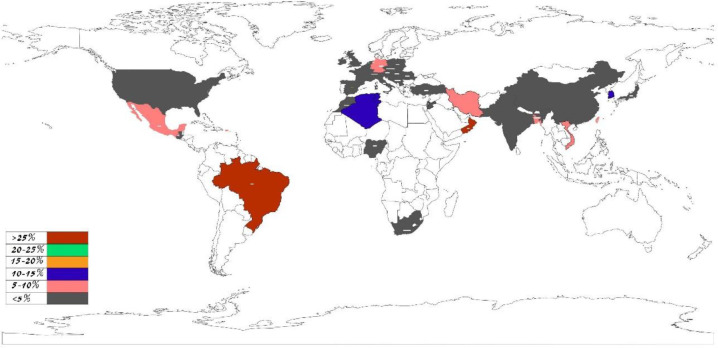
Geographical distribution of *MYO15A* mutations in patients with the autosomal recessive non-syndromic hearing loss who were GJB2 negative. The pooled frequency of *MYO15A* mutations was calculated for all countries, where data were available. Brazil and Oman had the highest rate of *MYO15A* mutation compared to other areas. Japan, Guatemala, Nigeria, and South Africa had a minimum of 2% mutation frequency

To determine the importance of this gene mutation in the development of HL in different parts of the world, the pooled frequency of *MYO15A* mutations was calculated for all countries, where data were available (Figure 4 and Table 2). The frequency rates varied from zero to more than 25% based on the included articles; for instance, in a study conducted in Turkey and two experiments in Iran, no mutation was found in the *MYO15A* gene (9, 15, 16). 

**Table 1:** Characteristics of included studies in the meta-analysis

			()	()	*GJB2*	*Myo15a* (%)#		*	
(34)	2001	Pakistan	100	112		9.8	Linkage analysis	Moderate	
(35)	2007	Turkey	115	41	41	4.8	Homozygosity mapping	Low	
(25)	2007	India, Pakistan, and Turkey		600	600	3.3	Linkage analysis	Moderate	
(36)	2009	Iran		40	34	5.8	Linkage analysis	Low	
(37)	2009	Tunisia	50	77	77	3.9	Linkage analysis	Moderate	
(31)	2010	Turkey	100	104	104	9.6	Autozygosity mapping	Low	
(38)	2010	Palestine	288	20	20	10	homozygosity mapping	Low	
(30)	2011	Turkey	100	49	49	9.9	Autozygosity mapping	Low	
(23)	2011	Israel, Palestine	240	11	11	18.1	NGS***	Low	
(16)	2011	Iran		37	31	0	Homozygosity mapping	Low	
-(39)	2012	Turkey and Iran	15	20	20	15	WES	Low	
(40)	2012	Iran	100	140	140	5.7	Linkage analysis	Low	
(26)	2013	Japan	144	98	68	8	NGS	Low	
(41)	2013	Korea	409	13	13	15.3	WES	Low	
(24)	2013	China	200	117	117	4.2	NGS	Low	
(42)	2014	Israel, Palestine	700	96	96	2	NGS	Low	
(43)	2014	Pakistan	89	30	14	28	Homozygosity mapping	Low	
(44)	2014	Germany	9	23	14	7	NGS	Low	
(45)	2015	Turkey		29	22	4.5	NGS	Low	
(4)	2015	Japan	269	854		1.1	NGS	Low	
(46)	2015	China	100	63	63	3.1	NGS	Moderate	
-(29)	2015	Iran		302	302	9.6	NGS	Low	
(47)	2015	Algeria	200	65	10	10	WES**	Low	
(48)	2016	Iran		25	25	4	Linkage analysis	Low	
(8)	2016	Turkey, Iran, Mexico, Ecuador, and Puerto Rico		160	160	7.5	WES	Low	
(48)	2016	Iran		25	25	4	Linkage analysis	Low	
(2)	2016	South Africa, Nigeria, USA, Tunisia, India, Iran, Turkey, and Guatemala	200	342	342	2	NGS	Low	
(22)	2016	Western Europe	6	131	131	3	NGS	Low	
(9)	2016	Iran		16	14	0	Linkage analysis	Low	
(12)	2016	Iran	100	30		3.3	Linkage analysis	Moderate	
(49)	2017	Japan	200	143	143	4	NGS	Low	
(50)	2017	Korea	32	28	25	8	WES	Low	
(51)	2017	Morocco		33	33	3	WES	Moderate	
(52)	2017	China		207	152	2.5	NGS	Low	
(53)	2018	Spain		50	50	2.2	NGS	Low	
(54)	2018	China		18	12	25	NGS	Low	
(55)	2018	Israel	110	47	13	15.3	NGS	Low	
(56)	2018	USA		33	23	4.3	NGS	Moderate	
(57)	2018	China		44	28	10.7	NGS	Low	
(58)	2019	Vietnam	117	87	55	7.2	NGS	Low	
(59)	2019	Pakistan	200	40	25	8	NGS	Low	
(60)	2019	Taiwan	128	41	41	4.8	NGS	Low	
(61)	2019	Iran		28	28	10.7	WES	Low	
(62)	2019	china	200	33	26	7.6	WES	Low	

*Quality assessment was done by the quality assessment checklist for prevalence studies, **Whole-exome sequencing, ***Next-generation sequencing, # The frequency of MYO15A mutations is achieved through dividing the number of patients with MYO15A mutation by the number of all nonsyndromic GJB2 negative patients

**Table 2:** The regional prevalence of *MYO15A* mutations in GJB2 negative ARNSHL patients

Country	Frequency of MYO15A mutations (%)
Brazil	52
Oman	30
Korea	10.5
Algeria	10
Mexico	7.5
Puerto Rico	7.5
Vietnam	7.2
Germany	7.1
Iran	5.8
Banglaka	5
Pakistan	4.9
Taiwan	4.8
Palestin	4.7
China	4.7
Turkey	4.4
United States	3.8
Israel	3.7
India	3.6
Europe	3.1
Moroccan	3
Tunisia	2.3
Spain	2.2
South Africa	2
Nigeria	2
Guatemala	2
Japan	2

## Discussion

According to the results of this meta-analysis of data from 44 studies conducted among 4176 GJB2 negative patients suffering from HL, there was a pooled global frequency of *MYO15A* mutation rate of 6.2%**,** that represents a prominent role for this gene mutation in hereditary HL. Nowadays, routine screening of *MYO15A* mutation is not performed in deaf patients due to its large size and having many exons (66 exons) (17). Also, methods like whole-exome sequencing, linkage analysis, and homozygosity mapping in consanguineous families were performed to detect *MYO15A* mutations, which are expensive, and this is a barrier to implementing them.

To the best of our knowledge, so far, few studies have synthesized the *MYO15A* mutation data in patients with ARNSHL worldwide. Nevertheless, in several studies, this gene was introduced as the second most crucial gene after *GJB2* as a cause of HL (2, 8, 10, 18). 

Many studies assayed the prevalence of mutations in the *GJB2* and *SLC26A4* genes among patients with ARNSHL in different parts of the world. They demonstrated that the prevalences of mutations in GJB2 and *SLC26A4* are about 15% to 25% and 2% to 12.6%, respectively, which are dependent on the region (19). Therefore, *MYO15A* is the third most important gene contributing to ARNSHL. Additionally, the frequency of *MYO15A* mutation is higher than *SLC26A4* mutations in several regions and has priority. 

Except for the frequency of common founder mutations in Brazil and Oman, *MYO15A* mutation frequencies in Algeria and Korea were higher in comparison to other areas (10%). Japan, Guatemala, Nigeria, and South Africa had the same minimum of 2% mutation frequency (Figure 4). A broad spectrum of *MYO15A* mutation was discovered around the world, 68.62% of which are missense mutations (20). Moreover, most of these mutations were reported in studies as novel variants, and only some were repeated in a few studies. For example, c.7395+3G>C was identified in Tunisian patients in two studies (21, 22) and 8183G>A was discovered in patients from three different countries, namely, China, Brazil, and Israel (18, 23, 24). In addition, 9478C>T was observed in Japanese patients in two studies and one Pakistani patient (4, 25, 26).

There are several founder haplotypes in *MYO15A* that are specific to a particular geographic zone, similar to *Val1400Met* (c.4198G>A) variation, which is found in about 50% of patients with ARNSHL in a region of Brazil and Turkey due to endogamous nature of these regions (18, 27). The haplotypes associated with *Val1400Met* in Brazil were different from those in Turkey. Additionally, in Oman, 30% of patients had a specific duplication mutation (c.1171_1177dupGCCATCT) (28). Two founder mutations, including p.Tyr1392Stop and p.D2720H (c.8158G4C) were reported in Iran and Pakistan, respectively (25, 29). Two variants of p.R1937TfsX10 (c.5809C>T) and p.S3335AfsX121 (c.9995_10002dup) were probably founder haplotypes in Turkey (27). 

The presence of these founder mutations in isolated populations might be useful to create a population screening for hereditary HL. Genotype-phenotype correlation between mutated region and severity of HL was reported in this gene.* MYO15A* gene mutations often cause severe to profound HL; nevertheless, the severity of deafness is moderate to severe only in patients with mutations in exon 2 (2, 21, 25, 30-33). 

These observations may reflect the presence of modifier factors. Most mutations in exon two were reported in the Middle East, such as Turkey, Iran, Pakistan, and Palestine. As a result, examining the second exon of *MYO15A* in patients in the Middle East, as well as patients with milder HL had the priority (63).

Several points should be considered in interpreting the results of our study. First, there is heterogeneity between the results of different studies due to using various mutation detection methods. Some methods are not able to identify all existing mutations. As a result, the prevalence of mutation may be underestimated. On the other hand, non-pathogenic variants discovered in patients with HL may be incorrectly considered mutations. 

*MYO15A* mutations cause the autosomal recessive form of HL; therefore, we extracted the exact number of autosomal recessive patients that did not have any *GJB2* mutations from each article and compared the frequency between them. In several studies, this number was not reported; accordingly, we omitted them from the analysis due to the lack of sufficient information. 

***Strengths and limitations of this study***

In the current study, only published studies were included, while those with negative results might be unpublished. Additionally, missing several relevant articles in the search process is possible. This study has several strengths, too. We used a manual search to have a comprehensive search strategy as much as possible. Heterogeneity was not observed in studies, which indicates the power of this study. 

Therefore, it is recommended for researchers to report their results clearly to simplify further meta-analysis. For instance, reporting the inheritance of HL or the number of patients with mutations in each gene is essential.

In most patients with ARNSHL, the *GJB2* gene was checked to identify the genetic causes of HL for only one exon. Although the genetic cause of deafness in many patients were a mutation in other genes, and due to the high cost of conventional sequencing methods, the genetic cause of the disease remains unknown in these individuals. 

## Conclusion

Based on the results of this the meta-analysis, after the examination of *GJB2* and *SLC26A4* in the patients with HL, checking the* MYO15A* gene is of paramount importance. Due to the large size of this gene, paying particular attention to geographic region is suggested, so as to perform the diagnostic tests properly. Moreover, if there is a dominant mutation in the region, initial assessment for the mutation will be recommended.

Otherwise, the preferred regions are the motor domain and sometimes the second exon of this gene. Accordingly, further studies considering the detailed data about the mutations, particularly in combination with the assessment of the other gene mutations to clarify the exact roles of the mutations in HL and the affected genes are recommended.
